# A palpable change in medical education post-COVID-19 pandemic

**DOI:** 10.11604/pamj.2023.46.70.37676

**Published:** 2023-10-25

**Authors:** Haitham Mohamed El Bingawi, Salah Eldin Abdel Hag Abdel Haleem

**Affiliations:** 1 Department of Internal Medicine, Sultan Qaboos University, Al Seeb, Muscat, Oman,; 2 Department of Pharmacology, Faculty of Medicine, University of Bahri, Khartoum, Sudan,; 3 Department of Pharmacology, College of Medicine, Al-Baha University, Al Baha, Saudi Arabia

**Keywords:** COVID-19, medical education, online teaching, faculty development, curriculum reform

## Abstract

COVID-19 has brought with it a global crisis, but it also highlighted many important take-home messages to educators that are worth considering and implementing for better future medical education. We highlight 5 of these messages; (1) redefining of what considered being a core in training, (2) better to be prepared for the future challenges of online learning, (3) re-envision faculty development practices, (4) attention shift from stethoscope to microscope, (5) extra-curricular scientific activities should not lag.

## Essay

During COVID-19 crisis peoples' lives have been affected in ways not seen in the whole of our lifetimes, likewise, medical education has been affected in ways depending on the country, institution, level, and/or individual student. The urge to report our experience, of lessons learned from this crisis, is like the urge to report a rare clinical case. Faculty and medical students were, suddenly, amid the global policy of social distancing and 'stay at home', a situation with zero face-to-face contact but with plenty of time for distant learning. Timetables, methods of teaching, content, and assessment had to be modified to utilize available resources. Global cessation of in-person teaching or clerkship amid the pandemic has been described by previous reporters [1,2]. In the present work, we are reporting the key lessons learned amid the COVID-19 pandemic dictated by the palpable changes in medical education.

**Redefining of what considered being a core:** upon discussing with many colleagues how they succeeded in the transition from face-to-face to online learning. Most of them responded that they first decided to review the content and answer the question of *what can be left behind without affecting the standards*. Most, if not all, realized that a considerable amount of theoretical content can be left behind safely. According to Hardens, the core contents are to be specified by the teacher or the subject expert, and they are [3]. Therefore, there is a big question mark over the amount of information we deliver to medical students which may not all be necessary. We argue the pressing need for critical revision of what is really considered to be core. This is not always observable to medical teachers and curriculum designers in the ordinary teaching days. We observed that the students' attendance of the scheduled online teaching was excellent, perhaps, better than that of the face-to-face classroom. In frequent discussions with students, many of them suggest the replacement of some of the traditional lectures with online recorded lectures or virtual classes. They argued that online lectures are more focused and to the point. It is not necessary that we follow these suggestions, but pertinent to change the way we conduct our traditional lectures. The lectures may need to be delivered in shorter sections and perhaps incorporate more interactive materials. We need to consider the profile of the current generation who likely benefits from a structured but also more interactive learning experience [4].

**Online learning is inevitable in the future of medical education:** it is obvious that tomorrow´s doctors need knowledge and skills different from the current ones [5]. This experience has opened my eyes to the numerous benefits of online education, which makes learning convenient and flexible.

**Furthering the role of simulated activities:** simulated virtual, theoretical, and practical, classes are notions central to distant learning. Do they fit the needs of medical students? We have learned that most students are more open, and interactive, to these activities. This is justifiable by the fact that these innovative methods provide a range of choices for variable cohorts of students such as shy students, nocturnal, or own-pace self-learners. This does not signal a total future shift for these methods but their incorporation as an integral significant part of teaching in medical schools. This shift towards simulated learning modalities is encouraged by the reduced costs and time requirements compared to conventional wet-laboratory or bedside teaching [6]. However, since students’ acceptance and interaction with these innovative methods are variable, redistribution of students along the ladder of excellence should be anticipated. Learning resources is yet another issue. The crisis has augmented the realization that traditional libraries are giving way to digital ones. A lesson learned is that medical students should be provided with free access to authentic and copyrighted sources of knowledge. Furthermore, students should be trained to evaluate and critically appraise scientific information [7]. This was clearly obvious by the observation that scientific reports about COVID-19 were updated in a way that they were superseded by newer evidence-based reports by the day.

**Adopting dynamic study schedules:** timetables for online teaching should be designed in a flexible and dynamic manner. It´s observed that students had the opportunity to face experiences not usually offered in the regular curriculum, for instance, reviewing the curriculum materials at the time they select, creating a study schedule that suits their lives, more excitingly, encountering a broad, flexible, and dynamic timetables. It is, perhaps, more convenient for far-living students to attend virtual classes than attending face-to-face classes. Students do not need to worry about missing traditional classes, especially in conditions like bad weather or personal commitments. Furthermore, this flexibility may carry a huge benefit for non-traditional students. Critical to the discussion, online learning is not to replace traditional methods but rather a complement to it in a blended approach [8]. Although online education provides benefits and flexible training experience to students, it could place a huge time constraint on the already busy health professional staff. Universities as well as health facilities should secure some time for their healthcare educators to develop the skills necessary for producing online resources and acquiring new teaching styles [9]. Notably, there is a need for careful planning, meticulous archiving of resources, and follow-up of student attendance. One last summary point, the environment in which medical students are going to be trained soon will be very different from that of today. Online learning, unquestionably, will be in the center of any shift; for that reason, it is better for medical educators to be prepared for the inevitable challenges of this mode of teaching and assessment and they need to be prepared to innovate.

**Re-envision faculty development practices:** the quality of the faculty members is the most important resource of any medical school. As the world experiences the cascading effects of COVID-19, medical schools have dispersed online resources. The success, or failure, of this transition depended mostly on faculty members, the quality of their online resources, and on their interests and expertise. Therefore, for better academic validity and to manage such a transition or any out-of-hand situation in the future, medical colleges should invest more in the development and training of their faculties. On the other hand, such a transition to online education may also make some faculty members vulnerable to new contract conditions. For example, part-time agreements or on-demand service offers, may not be eligible to participate in faculty development programs [10]. It's perhaps fitting to conclude this point by saying that universities should not underestimate the importance of providing full academic and social support to their faculty members, this will return positively in their performance during a similar crisis. However, the challenges of faculty development are great, faculty members need to understand the whole picture before being asked to develop new strategies of teaching or assessment in this very new teaching environment. The questions that should be raised by medical institutes are as follows: what training program should be offered? How frequent? What strategies to implement? What follow-up support is to be offered? How to evaluate the faculty performance during similar crises and to what extent? Should the training be at the college level or individual level? How to motivate the faculty members?

**Attention shift from stethoscope to microscope:** the COVID-19 pandemic highlights the urgent need for physician-scientist and science-backed research. It has, perhaps, changed how we look at basic sciences. It's worth noting that, despite the current trend of integration of basic and clinical sciences, most medical schools are far from achieving the ideal merger between the two. Still, clinical sciences flourish over basic sciences. As a result, it´s not a reasonable expectation to see medical students recognize the value of basic sciences for their future clinical practice. Many studies that looked at the career choice of medical students upon graduation document the majority prefer clinical sciences [11,12]. The fact is that basic sciences are the bridge to the future physician-scientist [13]. Physician-scientists have been a driving force in biomedical research; they have altered the history of medicine and contributed to 37 percent of Nobel Prizes in Physiology or Medicine [14,15]. The answers to the difficult questions of COVID-19 will, most likely, come from a basic scientist or a physician-scientist. Therefore, the timing is just appropriate to re-evaluate the role of basic science in the medical school curriculum. Curricula should be tailored to promote the development of both clinicians and scientists. This could be achieved by reviewing the way basic sciences are taught and integrated into the curriculum and, more crucially, by identifying factors for the decline of their popularity among students. As argued by Mukesh *et al*. we need to ensure that the brightest young doctors can contribute to further advancements in their field [14,15].

**Extra-curricular scientific activities should not lag behind:** during the current COVID-19 crisis, seminars, webinars, and many other types of presentations were successfully conducted by students and faculty. On the other hand, it has been observed that most scientific conferences that were previously scheduled to take place have been postponed due to the pandemic. It has been observed that governmental, inter-governmental, organizational, and commercial meetings were successfully held online. Scientific meetings, including international conferences, shouldn't have lagged. A future lesson for every preparatory organizing committee of these events is to prepare for the possible shift to online proceedings. In fact, this is applicable even under normal circumstances. The effectiveness of logistics will enable more faculties to attend these events. Furthermore, these meetings could be held more frequently if need be.
